# Intra-thoracic fat volume is associated with impaired myocardial function and adverse left ventricular remodeling in patients with known or suspected coronary artery disease

**DOI:** 10.1186/1532-429X-18-S1-P83

**Published:** 2016-01-27

**Authors:** Anna Schmidt, Alessandro Satriano, Kate Fenwick, Nowell M Fine, Bobby Heydari, Todd Anderson, Matthias G Friedrich, James A White

**Affiliations:** 1 Stephenson Cardiovascular Magnetic Resonance Imaging Centre, Libin Cardiovascular Institute of Alberta, Calgary, AB Canada; 2 grid.22072.350000000419367697Division of Cardiology, Department of Medicine, University of Calgary, Calgary, AB Canada; 3 grid.14709.3b0000000419368649Division of Cardiology and Radiology, McGill University, Montreal, QC Canada

## Background

Intrathoracic fat volume (ITFV) is a reproducible imaging biomarker and has been associated with coronary artery disease (CAD) and myocardial infarction. Relationships between ITFV and adverse left ventricular remodeling of the non-infarcted myocardium are unknown. We explored the relationship between ITFV and 4D myocardial strain-based markers of adverse remodeling in the non-infarcted myocardium of patients with known or suspected CAD.

## Methods

Forty-seven patients referred for known or suspected coronary artery disease were studied at 3.0 Tesla using a standardized CMR protocol. This was inclusive of sagittal black blood (HASTE) imaging of the thorax (fat quantification), short and long-axis cine imaging, and matched late gadolinium enhancement (LGE) imaging. Blinded visual interpretation of LGE images was performed to determine the presence of prior myocardial infarction. Image analysis was performed by a separate blinded interpreter (cvi42, Circle Cardiovascular Inc., Calgary).to determine LV volumes, mass and the segmental extent of LGE. Segmental strain measures were obtained using in house software (GIUSEPPE). All myocardial segments with ≥10% LGE by volume were considered to have replacement fibrosis and were therefore excluded from analysis.

## Results

The mean age was 57 ± 13 years and mean BMI 29.9 ± 6.2 kg/m^2^ (range 20.3 to 38.4 kg/m^2^). The mean LVEF was 49 ± 20%. Prior ischemic injury was identified in 25 (53%) patients, leading to the exclusion of 193 (26%) of 752 segments from analysis. Amongst all subjects, elevation in ITFV was associated with a significant reduction in peak circumferential strain (r = 0.482, p = 0.001), longitudinal strain (r = 0.432, p = 0.002), radial strain (r = -0.387, p = 0.007), minimum principal (r = 0.477, p = 0.001) and maximum principal strain (r = -0.456, p = 0.001). Following adjustment for infarct size higher ITFV was similarly associated with global markers of adverse remodeling, including LVEDV index, LVESV index, and LV mass index (p < 0.05 for all). The clinical variable of adiposity, Body Mass Index (BMI), failed to show associations with strain parameters or with LV volumes. BMI did show significant correlation with the LV mass index. Subgroup analysis of subjects with and without LGE evidence of prior MI demonstrated worsened strain metrics in the MI group, including peak circumferential strain (-8.6 ± 3.9% vs -11.1 ± 3.2%, p = 0.017), longitudinal (-7.7 ± 3.3% vs -9.9 ± 3.0%, p = 0.020) and minimum principal strain (-12.1 ± 4.3% vs -14.9 ± 3.6%, p = 0.020). Significant associations between ITFV and strain measures were maintained in each subgroup.

## Conclusions

Among patients with known or suspected CAD intra-thoracic fat volume is associated with reductions in myocardial strain in the non-infarcted myocardium. These findings suggest ITFV to be a potentially important marker of adverse ventricular remodeling that is not appreciated by conventional clinical measures of adiposity.

